# Beyond small molecules: targeting G-quadruplex structures with oligonucleotides and their analogues

**DOI:** 10.1093/nar/gkab334

**Published:** 2021-05-12

**Authors:** Enrico Cadoni, Lessandro De Paepe, Alex Manicardi, Annemieke Madder

**Affiliations:** Organic and Biomimetic Chemistry Research Group, Ghent University, Krijgslaan 281 S4, B-9000 Ghent, Belgium; Organic and Biomimetic Chemistry Research Group, Ghent University, Krijgslaan 281 S4, B-9000 Ghent, Belgium; Organic and Biomimetic Chemistry Research Group, Ghent University, Krijgslaan 281 S4, B-9000 Ghent, Belgium; Organic and Biomimetic Chemistry Research Group, Ghent University, Krijgslaan 281 S4, B-9000 Ghent, Belgium

## Abstract

G-Quadruplexes (G4s) are widely studied secondary DNA/RNA structures, naturally occurring when G-rich sequences are present. The strategic localization of G4s in genome areas of crucial importance, such as proto-oncogenes and telomeres, entails fundamental implications in terms of gene expression regulation and other important biological processes. Although thousands of small molecules capable to induce G4 stabilization have been reported over the past 20 years, approaches based on the hybridization of a synthetic probe, allowing sequence-specific G4-recognition and targeting are still rather limited. In this review, after introducing important general notions about G4s, we aim to list, explain and critically analyse in more detail the principal approaches available to target G4s by using oligonucleotides and synthetic analogues such as Locked Nucleic Acids (LNAs) and Peptide Nucleic Acids (PNAs), reporting on the most relevant examples described in literature to date.

## INTRODUCTION

### G-quadruplex structural features and properties

**Figure 1.**
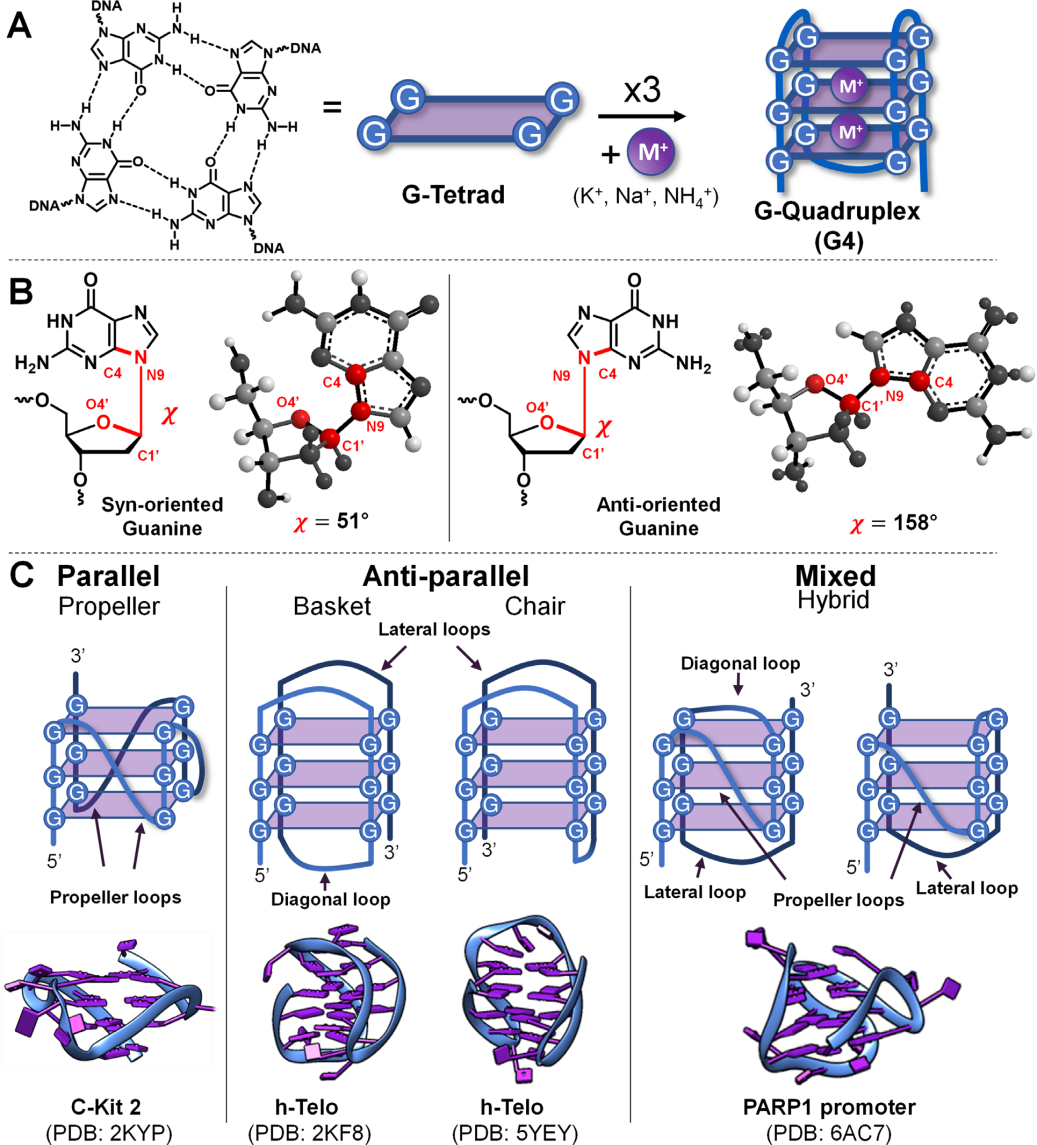
(**A**) Representation of the G-tetrad structure, here stabilized by a monovalent cation (M^+^), and G-quadruplex (G4) formation by stacking of three tetrads. (**B**) Illustration of *syn*- and *anti*-oriented guanines, the dihedral angle is highlighted in red. (**C**) G4 topology representations (top) and examples of their 3D structures (bottom).

The human genetic material mainly occurs as a double stranded DNA (dsDNA) duplex, held together through Watson–Crick hydrogen bonds between complementary base pairs (1). In 1962, Gellert *et al.* discovered the spontaneous aggregation of guanosine monophosphate into a square planar rearrangement (2). A similar structure was later found in telomeric G-rich DNA sequences and, from then on, became known as a G-quadruplex (G4) (3–5). This alternative, non-canonical structure can arise once four guanines recognize each other and form a planar structure (Figure 1A), the so-called G-quartet or G-tetrad, stabilized by Hoogsteen-hydrogen bonds, followed by the subsequent rearrangement of the G-containing sequence into a quadruplex structure due to stacking of two or more of such tetrads. These structures can be formed either intramolecularly, if only one strand containing at least four guanine runs of at least two guanines each (G-runs), is involved (intramolecular quadruplex). Alternatively, they can be formed in an intermolecular fashion, between two or more G-rich strands (intermolecular quadruplex) (6).

Additionally, a monovalent cation is needed to provide electrostatic stabilization and render G4-formation favourable under physiological conditions (7). Among the relevant cations available in a cellular environment, the highest contribution to quadruplex stability is observed for K^+^, due to its larger ionic radius which results in a lower solvation enthalpy and, as a consequence, in a lower energy loss as compared to Na^+^ (8). In addition, the ionic radius of K^+^ allows the cation to fit in between two G4 tetrads, coordinating the O6 of the eight guanines (9). Interestingly, the intracellular concentration of K^+^ in cells is higher (140–150 mM) as compared to Na^+^ (12 mM). Complexation in presence of some divalent cations, such as Sr^2+^ (10,11), Ba^2+^ (12), Ca^2+^ (13) and Pb^2+^ (14,15) is also reported, and such complexation abilities were exploited for the realization of metal sensing applications (16). In contrast, even when a stabilizing cation is present in solution, a G4 structure can be disrupted by the presence of Mn^2+^, Co^2+^ and Ni^2+^ divalent cations (17).

The guanines participating in the G-tetrad formation can be found in two different conformations, depending on the dihedral angle between the O4′-C1′ bond of the ribose and the N9–C4 bond of the guanine: *syn*-guanine, where the base is oriented over the furanose ring, and *anti*-guanine, in which the base is oriented away from the sugar (18) (Figure 1B). While in regular dsDNA and ssDNA the guanines preferentially adopt an anti-conformation, in DNA G4-structures *syn*-oriented bases, although less stable, can be found.

Based on this orientation and on the disposition of the loops (part of the sequences between G-runs and not involved in tetrad formation), G4s can fold into three main topologies: parallel, anti-parallel or hybrid (depicted in Figure 1C). Parallel (or propeller) structures are characterized by the presence of three propeller-type loops, which connect the upper and lower tetrad, and contain only guanines in anti-conformation. Anti-parallel G4s can be either classified as chair conformations, if they contain only lateral loops (connecting adjacent guanines of the same external tetrads), or basket conformations, if they contain both lateral and diagonal loops (connecting opposed guanines of the same external tetrads). Finally, mixed or hybrid conformations can contain all three different types of loops: diagonal, lateral, and propeller. In contrast to parallel structures, anti-parallel and mixed conformations, can contain both *syn*- and *anti*-oriented guanines (19,20).

Various studies have revealed that the length and the composition of the loops have an impact on the stability of the quadruplex. In general, short loops are preferred in terms of stability and represent the highest population, although unusually long loops are present in some sequences of interest (21,22).

The formed topology mainly depends on the nature of the sequences involved, the stabilizing cation and the ionic strength (20). Important G4-forming sequences have been shown to adopt different topologies in case of variations in one or more of these conditions (23,24). As an example, for the human telomeric 22mer G-rich repeat (h-Telo) an intramolecular parallel structure and a hybrid structure have been found in presence of K^+^ (25,26), while a basket topology was observed in Na^+^ containing buffers (27). In addition, the presence of G4-binding small molecules (see section 1.2 for more elaborate information on such G4-binders) can also induce a conformational switch between the different topologies (28,29). Besides genomic dsDNA and ssDNA structures, G4-forming sequences can be found in RNA (30). Indeed, the first example of a quadruplex occurring in a RNA oligonucleotide was discovered in 1991, in a 19mer sequence from *Escherichia coli* (31). Due to the structural difference between DNA and RNA, mainly residing in the presence of the hydroxyl group in 2′ of the ribose ring, the quadruplexes formed in the latter case exhibit peculiar features. The hydroxyl-group in position 2′ disfavours the *syn*-conformation of the guanine. This can be attributed to the presence of the OH, which induces the 3′ endo ribose puckering, limiting the possible dihedral angles in which guanine can be found (32). As a consequence, RNA quadruplexes are more prone to fold in parallel topologies, although anti-parallel RNA quadruplexes are reported to exist under specific conditions (e.g. sequences containing 8-bromoguanosine) (33). Additionally, the conformations adopted are less dependent on the experimental conditions (e.g. type of cation, presence of ligands), reducing the variety of G4 RNA structures found as compared to DNA ones (34).

### Localization of G-quadruplexes in the human genome and transcriptome and significance of G4-targeting

Since their first discovery, thousands of putative G4-forming sequences have been identified, investigated and their presence in the human genome confirmed (35,36). Algorithm-based genomic studies demonstrated that the most abundant G-rich regions in the human genome are the TTAGGG tandem repeats of telomeres (37), the single-stranded overhangs found at the extremities of each chromosome, whose role is protecting the cell from genetic information loss at the end of each replication cycle (38). In cancer cells, telomerase, a reverse transcriptase normally active only in germinal cells, can extend these regions, inducing continuous cell proliferation (39). Stabilization of telomeric G4s with small molecule binders (e.g. telomestatin, BRACO-19) allows to either directly inhibit telomerase elongation or to block DNA replication (40,41). Recent studies showed that telomerase can unfold and extend telomeric regions and the presence of ligand-stabilized G4s can account for a reduction of telomerase activity to 65–70% (42).

Another key area for localization of G4-forming sequences was found in the promoter region of proto-oncogenes involved in important carcinogenic pathways. Hence, in case of dysfunctions related to neoplastic growth, the induction of G4s in these promoter regions can be used for transcription regulation, as it was reported that the formation of a stable DNA-G4 inhibits transcription initiation, blocking RNA polymerase and leading to a downregulation of the gene expression (35). More recent studies, however, suggested a more complex involvement of G4s in gene expression. More specifically, the abundance of G4 structures in regulatory regions of human chromatin and evidence of an increased gene expression associated with the enhanced G4 folding of these sequences underline their role in the positive regulation of gene expression (43). Additional studies have shown that G4s can bind to transcription factors at gene promoter levels. It was suggested that these G4s structures may act as a docking site for transcription factors (44). An example can be found in the *KRAS* promoter region, which contains a nuclease hypersensitivity site, partially overlapping with a G4-forming sequence. When the G4 structure is formed, the binding of MAZ transcription factor is favoured, leading to an upregulation of *KRAS* transcription (45). The first G4-forming sequence in promoter regions was discovered in the Pu27 sequence, located in the nuclear hypersensitivity element III of the *c-Myc* oncogene promoter (46), a transcription factor whose overexpression is linked to over-activated telomerase activity and cellular proliferation (39). Later on, the existence of G4s in other proto-oncogene promoter sequences was demonstrated, notably in the promoter regions of the vascular endothelial growth factor (VEGF) gene, involved in tumour angiogenesis (47); *c-Kit*, which encodes for a growth factor receptor tyrosine kinase and enhances cell proliferation and differentiation once it is over activated (48); *BCL-2*, encoding for an inhibitor of apoptosis-mediated cell death (49); *PARP1*, encoding for a nuclear enzyme involved in DNA-repair processes, cell-signalling and apoptosis (50); the aforementioned *KRAS*, encoding for a GTPase protein involved in numerous signal transduction pathways, for which overactivation is often associated with the onset of colorectal and lung cancer (51,52). Besides their role in transcription regulation, quadruplexes have more recently been suggested to play a role in regulating DNA replication. Indeed, such structures can be formed during double-strand separation of the DNA by the helicase enzyme and have been shown to be involved in replication stalling (35). Moreover, the formation of quadruplexes plays a role in epigenetic modulation. DNA methyl-transferases have a prominent binding preference for G4 over dsDNA, and such binding is associated with an inhibition of DNMT-1 activity, resulting in DNA hypomethylation (44).

At the RNA level, G4-tracts are ubiquitously present, which entails that they can be implicated in the regulation of important biological processes, such as translational regulation, transcription termination, 3′-end processing (53). Transcription of the C-rich DNA present in the telomeric repeats of the human genome leads to the formation of the so-called ‘telomeric repeats containing RNA’, known as TERRA, a remarkable and widely studied example of a RNA–G4 (54). The study of a bimolecular RNA quadruplex formed by the association of two homologous TERRA strands has significantly contributed to understand the structural properties of RNA–G4s (55). Just like its ‘genomic counterpart’, TERRA has a role in telomer regulation with various mechanisms proposed, such as through the binding to shelterin, a protein complex implicated in the regulation of the telomeric ends (56,57), and through the epigenetic modulation of histone methylation at telomers (58). In mRNA, G4s are found abundant in the 5′ UTR regions, suggesting a role in the regulation of the translation initiation, once folded into quadruplexes (59). The first example of translational inhibition driven by G4 formation in mRNA was reported for the NRAS proto-oncogene, which translates to a protein implicated in cell differentiation and proliferation (60). It was more recently found that during DNA transcription, also hybrid DNA:RNA G4 structures can be formed which have been assigned a role in translation termination (61). Besides their occurrence in mRNA, RNA–G4s were recently suggested to play a role in the maturation of micro-RNA (miRNA) precursors and in mature miRNA, suggesting the possibility to influence miRNA maturation using RNA–G4 stabilizing ligands (62,63).

This widespread, but tactical, localization in crucial areas of the genome and the role of G4s in gene expression and biological process regulation, together with the first evidences for *in vivo* G4-folding (64,65), underscores the need for new approaches to uncover the high potential of G4-targeting strategies and justifies the high demand for development of G4-stabilizing ligands. The existence of DNA-G4s in the telomeres and promoter regions of oncogenes, and the proposed involvement of RNA–G4s in important biological processes, such as gene translational arrest, mRNA processing, mRNA localization and even in the non-coding RNAs, very well indicates the importance of targeting these structures (66,67). It is worth mentioning that G4s are widespread structures in nature since G4-forming sequences have been found not only in non-mammalian animals, but also in plants, fungi, bacteria, and even in the genome of viruses of the human viriome, such as HIV (68–72). Due to the enormous research efforts resulting from the recent pandemic, putative G4-forming sequences have recently been found in the SARS-CoV-2 genome (73,74). Although research in this field is still only incipient and controversy exists, these findings, together with preliminary experiments *in vitro*, may illustrate the potential of a G4-targeted therapy in this context. (75).

### Bringing G-quadruplex targeting ligands towards the clinic: a matter of selectivity?

To date, numerous ligands able to bind and stabilize the G4-architecture have been reported. Most of them feature planar aromatic rings that enable aromatic π-π stacking with the G-tetrads and positively charged residues that exert electrostatic interactions with the negatively charged phosphate groups of the DNA-G4 (76–81).

In the early works describing small molecules able to target G4, the main issue was a low sequence-selectivity over dsDNA. Now that the design of G4-ligands able to discriminate quadruplexes from dsDNA is more consolidated (82), the attention moved to the discrimination among structures with different topologies and an increasing number of small molecules featuring this desired selectivity has been reported (83). Although some ligands preferentially bind structures with a specific conformation, they are not able to recognize a specific sequence and can potentially bind all G4-structures in the genome exhibiting a similar conformation. In this context, the main challenge for the drug-designer resides in the structural similarities among these quadruplexes. In addition, when it comes to long G-rich sequences, featuring a large number of consecutive G-runs, there is a chance that the sequence can fold into multiple possible quadruplexes with the participation of different guanines (G4-isoforms), eventually adopting different topologies. These structures can be mutually exclusive or may be in equilibrium within each other (84–86). HIV-1-LTR represents a well-documented example of a G-rich sequence adopting multiple quadruplex-folds and is characterized by the presence of four different and overlapping G4-forming regions, named LTR-I to IV. Since G4-formation is correlated with a decrease in viral activity, this represents an extremely interesting target from a therapeutic point of view (87). While G4 structures formed within LTR-II and LTR-III are predominant in relevant cellular conditions, LTR-IV, which has been suggested to be a modulator of G4 formation in the promoter region, requires stabilization through a ligand (85,88).

Considering that most ligands still lack topology-selectivity and are not able to discriminate among specific sequences, the development of topology- and sequence-specific targeting methodologies has been identified as key to translate G4-targeting strategies to the clinic. However, the identification of sequence-selective small-molecule ligands requires careful design and considerable synthetic efforts (83) and is characterized by the need for recognition elements that enable sequence-specific targeting.

It is important to remember that, in cancer therapy, the successful application of small drugs relies on the strong dependence of the target cells to specific, over-activated, pathways, a phenomenon commonly referred to as ‘oncogene addiction’ (89). Therefore, the use of small molecules has stronger effects on malignant over regular cells. Unfortunately, also healthy cells that display a fast turnover rate (e.g. gut and skin epithelial cells), can be heavily affected by such non-specific drugs. In addition, this concept is not applicable in other contexts, such as anti-viral therapies, or the study of the effects of targeting a specific sequence in the genome or transcriptome.

The use of oligonucleotides (or analogues) is an alternative solution to overcome the lack of selectivity connected to small molecule-based approaches. Since G4s are oligonucleotide-based structures, the high specificity of the base-pair recognition can be exploited. Beside the quartets or tetrads, which are the ‘fixed’ elements of the quadruplex, there are other characteristic elements, which vary between each structure: the loops and the flanking regions. These elements ensure that each quadruplex is unique within the whole genome and can be exploited as key target in oligonucleotide (or derivatives)-based approaches.

**Figure 2.**
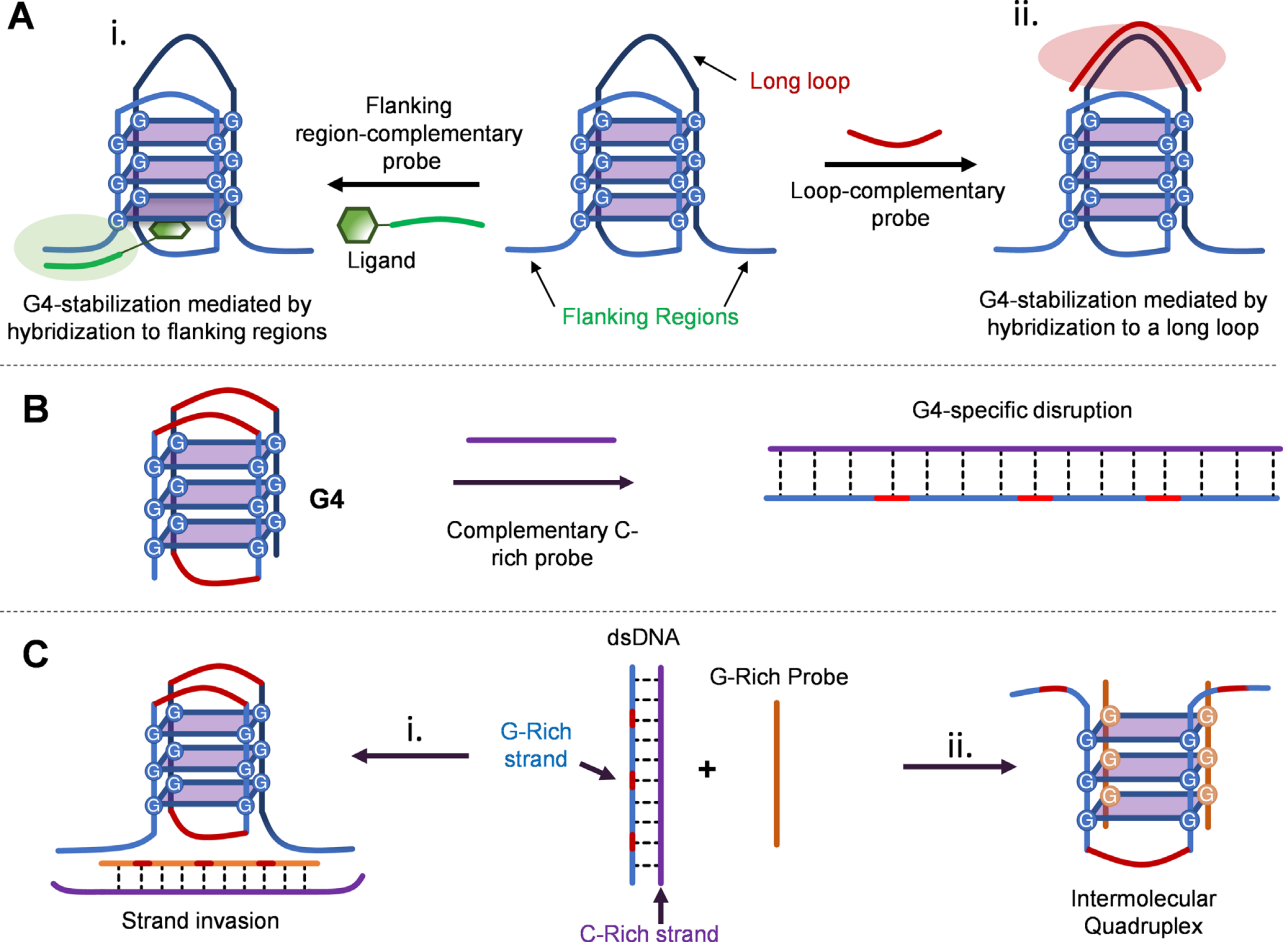
Overview of sequence-specific methodologies discussed in this review for targeting G4-forming sequences using synthetic oligonucleotides or mimics thereof. (**A**) Targeting flanking regions (i) and loops (ii). (**B**) Sequence-specific G4-disruption using C-rich probes. (**C**) G-rich synthetic probes for strand invasion approaches (i) or intermolecular quadruplex formation (ii).

Similarly to small-molecule binders, oligonucleotides can interact in several ways with their target, either inducing, stabilizing or disrupting the G4-formation (90). For example, 5′- and 3′-flanking regions and loops (if sufficiently long) can be exploited to allow targeting the desired quadruplex and bringing a ligand in close proximity to the desired G4-forming tract (Figure 2A). An alternative approach consists in aiming at the sequence-specific disruption of a G4, using cytosine-rich (C-rich) probes designed to hybridize to the target (unfolded) sequence (Figure 2B). Finally, guanine-rich probes can help in inducing intermolecular quadruplex formation (Figure 2C, right) as well as in separating the complementary C-rich strand in a strand-invasion-based approach (Figure 2C, left), facilitating quadruplex formation. In this review, after a brief description of the different recognition elements that can be employed, we will provide more insights into how these can interact with their target G4, describing specific requirements and advantages for each mode of interaction.

## OLIGONUCLEOTIDES AND ANALOGUES AS RECOGNITION ELEMENTS

**Figure 3.**
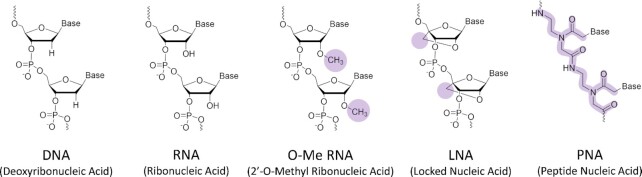
Structural comparison of nucleic acids and their analogues with applications in selective G4-targeting. The differences between DNA/RNA and the respective analogues are highlighted.

Because of their inherent sequence selectivity provided by the specific base-pairing recognition, the use of oligonucleotides can offer an important advantage over the mere use of small molecules for selective G4 targeting (91). However, for natural oligonucleotides, clinical applications remain limited by different factors, including nuclease-mediated cleavage of the phosphodiester bonds, unfavourable pharmacokinetics, and sub-optimal complex stability. In order to overcome these limitations, several typical structural elements of the classical oligonucleotides have been modified: the phosphate backbone (e.g. phosphorothioates or peptide nucleic acids), the pentose sugar (e.g. locked nucleic acids or 2′-*O*-methyl ribonucleic acids), and the nucleobases (see Figure 3) (92,93).

2′-*O*-Methyl ribonucleic acids (2′-*O*-Me RNAs) are a class of oligonucleotide analogues deriving from the methylation of the hydroxyl in position 2 of the ribose ring, which represents the only structural difference with RNA. The 2′-*O*-methyl group is a naturally-occurring modification, implicated in certain cellular processes involving ribosomal and nucleolar RNAs (94,95). When compared to the non-methylated counterparts, 2′-*O*-methylation confers higher resistance to nucleases, while guaranteeing high affinity to complementary RNA targets and similar properties, such as solubility, (96,97).

Locked nucleic acids (LNAs), reported by Singh *et al.*, represent a widespread type of nucleic acid analogues (98). LNAs preserve high structural homologies with the naturally occurring nucleotides: the phosphate and the ribose sugar are still present in the backbone, ensuring good water solubility. The most evident structural difference with respect to DNA oligos is the presence of a 2′–4′ methylene linkage that renders them resistant to nuclease-mediated degradation (99). This bridge also pre-organizes the single stranded LNAs into helical structures with enhanced stacking interactions which results in an exceptionally high thermodynamic stability and affinity towards complementary nucleic acids (100,101). In contrast to other oligonucleotide analogues, LNAs are still recognized by some enzymes, e.g. when hybridized to their RNA targets, RNAse-H can still induce RNA strand cleavage (102).

In contrast to LNAs, peptide nucleic acids (PNAs) display large structural differences when compared to natural DNA or RNA. First reported by Nielsen, these analogues are characterized by the presence of a neutral polyamide backbone, to which nucleobases are attached through methylene carboxylic linkers (100,103). The lack of negative charge has an important consequence on the DNA:PNA duplex stability. Indeed, the absence of electrostatic repulsion between the two backbones renders the formed complexes highly stable. Additionally, DNA:PNA duplexes display a more significant reduction of melting temperature when base mismatches are present, as compared to natural DNA:DNA duplexes (104). Another consequence of the uncharged backbone is that the stability of the resulting complexes is scarcely affected by variation of the medium (solvent, ionic strength and ion composition, pH) (105). Additionally, they exhibit high chemical stability combined with a natural resistance to enzymatic degradation by nucleases and proteases, not being recognized by the enzymes due to the unnatural synthetic backbone (106,107). The higher thermodynamic and enzymatic stability as well as the affinity of oligonucleotide analogues explains why they are preferably used in this context over the regular oligonucleotides.

The list of nucleic acid analogues is certainly more extensive, including, amongst others, morpholino oligonucleotides, phosphorothioates, phosphoramidates and other 2′-modified ribose oligonucleotides, such as *O*-methoxyethyl RNAs. However, unlike for PNA, LNA and 2-*O*-Me-RNA, no applications in selective G4-targeting have been reported to date for these derivatives.

## INDUCTION OF INTERMOLECULAR QUADRUPLEX FORMATION

The use of synthetic and modified oligonucleotides as recognition elements for specific DNA or RNA sequences adopted in classical antigene and antisense approaches, respectively, can be further extended to the sequence-selective targeting of G4s. As we have discussed before, there are various alternatives for targeting G4 structures. One often exploited method consists of providing oligonucleotides featuring guanosine-stretches (multiple runs of two or more consecutive guanosines), which have shown to be able to invade existing G4s, thus inducing the formation of alternative intermolecular (bimolecular) quadruplexes. These newly formed structures have been shown more effective at inhibiting DNA-polymerase-based extension as compared to the original, canonical, DNA intramolecular quadruplexes (108). As a direct consequence of the G-rich nature of these synthetic probes, they often show reduced solubility and a tendency towards G4-aggregation. This is a particular drawback when using PNA probes, although they are still the preferred oligonucleotide analogues for this application owing to the high stability of the resulting complex.

This kind of G-rich oligonucleotides and analogues are generally used to target existing G4-forming sequences, inducing the formation of more stable intermolecular quadruplexes (section 3.1, ‘targeting regular G-quadruplex forming sequences’). Applications where a missing guanine residue or entire guanine stretches are supplied to ‘complete’ the tetrads, which is necessary for the formation of a stable G4 structure, are also discussed (section 3.2, ‘Targeting defective Quadruplex sequences’).

### Targeting regular G-quadruplex forming sequences

The formation of G4s under physiological conditions is not always straightforward. Indeed, when the DNA is in its double stranded form, and the G-rich sequence is paired with the complementary C-rich strand, the formation of a quadruplex is discouraged as the double-stranded structure exhibits higher stability. The use of G4-binding ligands was then exploited to provide extra stabilization to the G4s, therefore shifting the equilibrium toward the formation of these structures. Artificial DNA analogues can be exploited to play the same role in a sequence-selective manner, but only if the resulting hybrid structure features a higher stability than the competing dsDNA structure.

**Figure 4.**
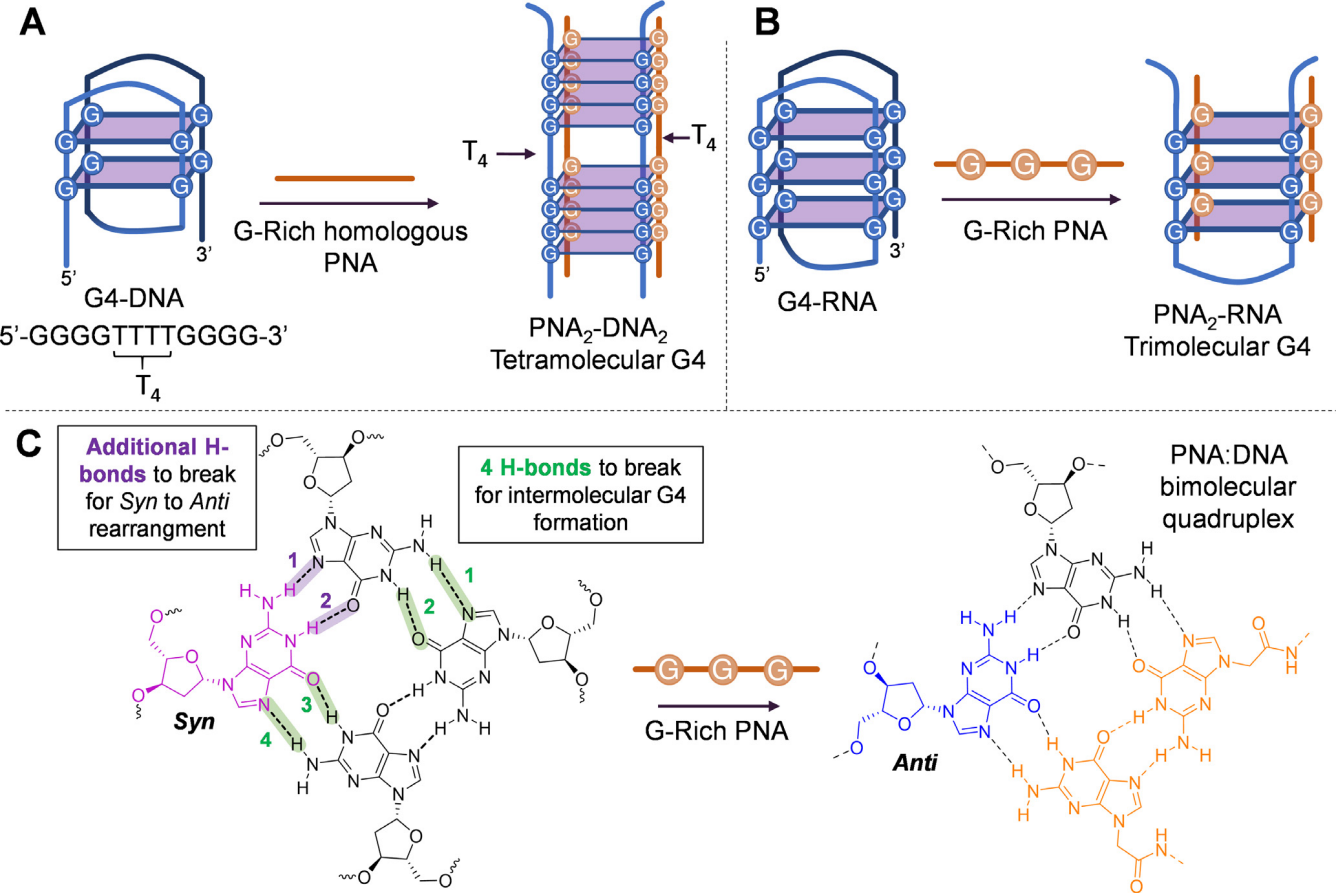
Targeting G4 by inducing the formation of intermolecular quadruplexes. (**A**) Scheme, based on the work by Armitage *et al.* (109), illustrating the rationale of the approach. (**B**) Targeting of RNA–G4 with PNA_2_:RNA trimolecular quadruplex formation, illustrated by Gaynutdinov (110). (**C**) Cartoon representation of the hydrogen bonds to be broken to induce intermolecular quadruplex formation. Based on the work by Roy *et al.* (113).

The ability of a PNA to form an intermolecular quadruplex with target DNA was first reported by Armitage and co-workers, who studied the formation of a tetramolecular PNA_2_:DNA_2_ complex in solution, using *Oxytricha Nova* telomere as model (109). The natural G4 structure is disrupted by the hybridization with the PNA strands, followed by the formation of a hybrid tetramolecular structure characterized by a remarkably high thermal stability that is less affected by ionic strength as compared to the DNA–G4 intramolecular quadruplex (Figure 4A). This can be attributed to the incorporation of the neutral PNA backbone into the G4 complex, which reduces the electrostatic repulsion induced by the phosphate groups. Using the same target sequence, the formation of PNA_3_:DNA tetramolecular quadruplex was later on demonstrated by another group (110). This approach was later extended to the invasion of RNA-G4s. Using a homologous short PNA probe, they reported on the formation of a stable PNA_2_:RNA intermolecular quadruplex, proposing a variety of possible structures. Formation of these hybrid G4s at low nanomolar concentration was demonstrated, and it was shown that the results do not depend on PNA orientation (parallel or anti-parallel). This suggests a lack of interaction in the loop region and implicates that the composition of the loop bases of the targeting probe can in principle be changed, without affecting its ability to induce intermolecular quadruplex formation (111,112).

When targeting G4 structures, the specific conformation is a crucial factor to consider. Indeed, for intermolecular G4 formation, the targeting of anti-oriented guanines is simpler from a thermodynamic perspective. In this case, the targeting probe can in fact invade the G-tetrads, splitting up the quartets by breaking four Hoogsteen hydrogen bonds. If the targeted quadruplex contains syn-oriented guanines, in addition to the four hydrogen bonds that need to be interrupted to perform the invasion, an additional two hydrogen bonds need to be broken to allow the rearrangement of the syn-guanines into the preferred anti-conformation for intermolecular quadruplex formation. As a consequence, G4s displaying a parallel conformation, which only contains anti-oriented guanines, are easier to invade. This concept, demonstrated by Roy *et al.* employing PNA probes (Figure 4C), can in principle be applied to other oligonucleotide analogues (113). Therefore, although literature reports are not yet available, we speculate that RNA–G4s, which are always found in a parallel conformation, thus exclusively featuring anti-oriented guanines, would be generally easier to invade as compared to DNA ones.

Loop composition is also an important factor that can influence the outcome of a targeting approach. The Armitage group reported, for the first time, the use of a short PNA oligomer to target a long-looped G4 containing a 17mer central loop. The PNA used was able to form complexes with exceptionally high stoichiometry (PNA_6_:DNA intermolecular quadruplex) (114), which was possible due to the length of the targeted sequence. Although examples of long-looped G4s have been reported (115), short-looped quadruplexes are more likely to exist in the cellular environment (116,117), and this is a factor that researchers have to consider when translating these targeting methodologies from the bench to cellular applications.

**Figure 5.**
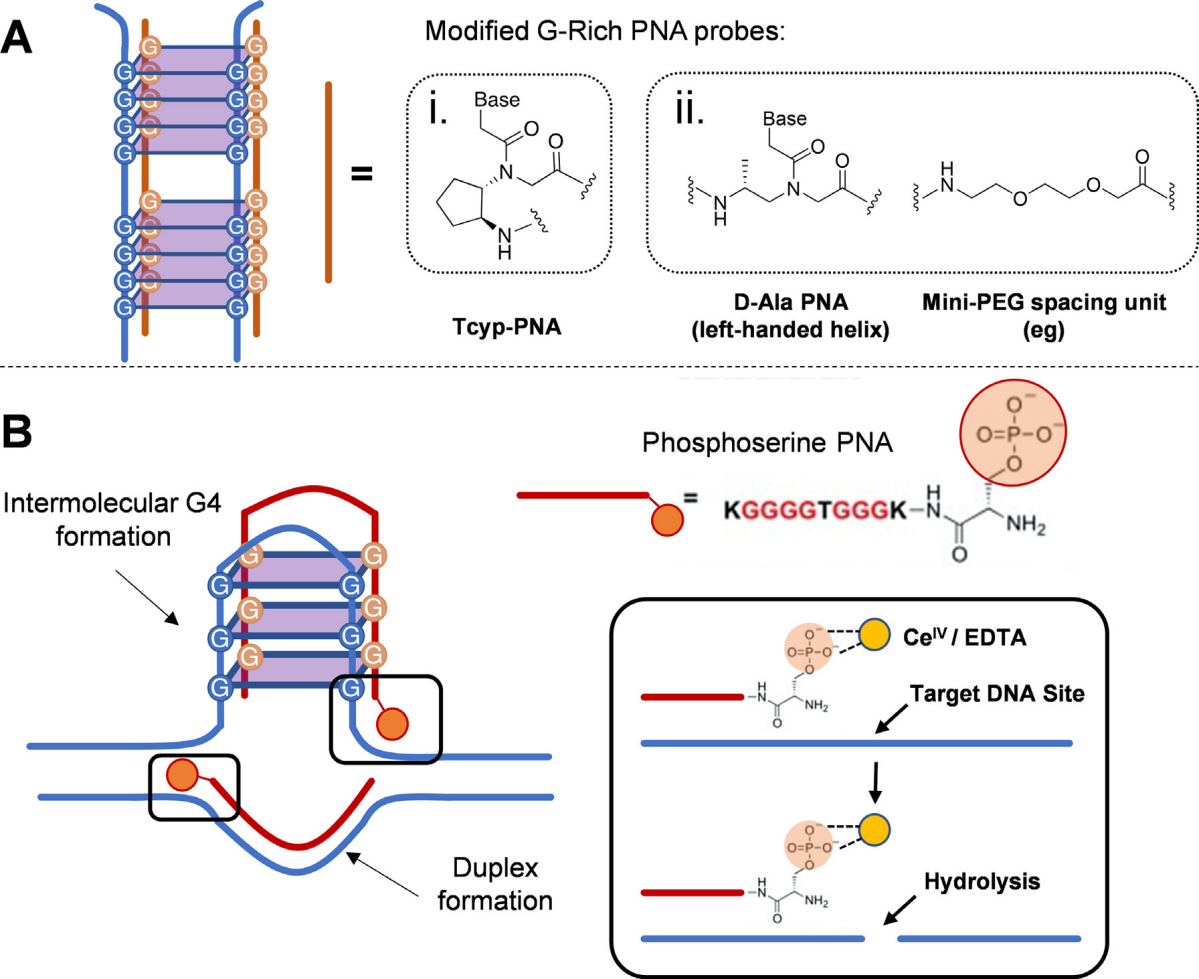
Overview of the PNA modifications described in this section employed to target G4 by inducing the formation of a PNA–DNA intermolecular quadruplex (**A**): i. Tcyp–PNA described by Englund *et al.* (118); ii. PNA modifications presented in the work by Armitage and colleagues (119). (**B**) The approach proposed by Komiyama and colleagues, describing the G4-formation-driven sequence-specific DNA scission; K corresponds to a lysine residue (122).

When using G-rich probes to induce intermolecular quadruplex formation, a competitive hybridization with the complementary C-rich strand can also occur, leading to the formation of an undesired double stranded PNA:DNA complex. In this context, PNA-backbone modifications can be conveniently exploited to improve the selectivity. Englund *et al.* showed that the incorporation of (*S*,*S*)-*trans*-cyclopentane (tcyp) units into the PNA backbone enhances PNA:DNA complex stability, with a more pronounced effect on intermolecular quadruplexes than duplexes (118). The introduction of four consecutive tcyp-modified guanine residues in the PNA structure, led to the formation of exceptionally stable PNA_2_:DNA_2_ tetramolecular quadruplex as compared to the use of the unmodified PNAs (ΔTm = +40.6°C and +14.3°C, respectively). On the other hand, the effect on the PNA:DNA duplex was more modest (Δ*T*_m_ = +5°C per modified unit as compared to the regular PNA). To further minimize the undesired competitive duplex formation, Armitage *et al.* proposed other modifications for enhanced quadruplex targeting, among which the use of a chiral, left-handed, γ-D-Alanine-modified backbone (γ-PNAs). Introduction of this modification induces a strong destabilization of the PNA:DNA duplex (Δ*T*_m_ = –26.0°C with respect to unmodified PNA) due to the induction of a left-handed helical pre-organization of the PNA probe that does not match the right handedness of the DNA helix. This helicity, on the other hand have a negligible impact on the stability of intermolecular quadruplexes. In addition, the G4 loop bases can be replaced by hydrophilic ethylene glycol linkers that reduce the number of potential Watson–Crick base pairs of the PNA:DNA duplex, without affecting the Hoogsteen hydrogen bonds of the intermolecular quadruplex (119). Combined together, these two modifications displayed a synergic effect, completely disfavouring the PNA:DNA duplex, which is not formed under experimental conditions, in favour of quadruplex formation. An overview of the discussed modifications is given in Figure 5A.

An elegant approach exploiting sequence-specific intermolecular quadruplex formation comes from Komiyama's lab report on the selective single stranded DNA hydrolysis operated by a Ce(IV)/EDTA complex (120,121). In this work, they used a single G-Rich PNA probe, decorated with a phosphoserine, designed to target both strands of c-Myc19 DNA. The designed probe was able to complex the c-Myc C-Rich strand and hybridize to the G-Rich strand, inducing the simultaneous formation of a PNA:DNA duplex and an intermolecular quadruplex. The phosphoserine included in the PNAs served as monophosphate group for the localization of the Ce(IV) complex allowing a G4-formation driven scission of the target DNA at the level of the PNA hybridization sites (122) (Figure 5B).

From the examples reported above, it is clear that considerable efforts have been spent in testing modifications of the probes to disfavour duplex formation. As most of these modifications concern the backbone, it appears logical that the use of PNA was preferred, due to the higher chemical flexibility of the polyamidic backbone, over the other available synthetic analogues. All these methodologies, although representing interesting approaches in the context of G4-targeting, still show a lack of complete sequence-specificity. To overcome this problem, one can foresee the use of longer probes, partially complementary to the up or down-stream flanking regions of the G4, provided the G-rich sequence for intermolecular quadruplex induction is located in close proximity to the targeted sequence. Besides the work from Komiyama, which encompasses an elegant application based on intermolecular quadruplex formation, further applications have not been explored in depth. Finally, most of the reported work focusses on DNA targeting, while it would be interesting to explore more in detail the RNA–G4 targeting, as it will not require the use of modified probes to avoid the hybridization of the probes to the C-rich counterparts.

### Targeting defective quadruplex sequences

As mentioned in the introduction, to form a stable quadruplex, four G-runs are required to elicit the self-assembly in presence of monovalent cations. However, not all G-rich sequences contain enough G-runs to form stable quadruplexes. To target sequences that cannot fold into G4s by themselves and require more runs, approaches were developed based on the delivery of an external oligonucleotide probe that, bearing the required missing guanine(s), has the ability to induce the G4-formation.

**Figure 6.**
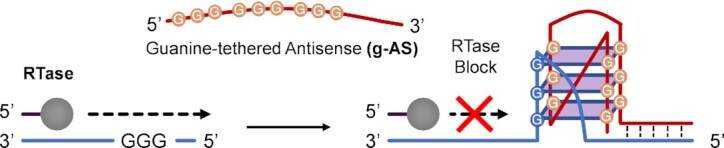
Targeting of guanine-deficient sequences. The figure shows the approach described by Nakatani *et al.*, as a general example (123). RTase activity is blocked by the formation of the intermolecular quadruplex, upon hybridization with the g-AS probe.

A first example can be found in the work of Nakatani *et al.* They reported on the use of an antisense DNA equipped with a G-rich tail, targeting the HIV-1 RNA sequence. When the DNA probe hybridizes to the target, the G-rich tail composed of only three G-runs is positioned in close proximity to a guanine stretch within the target, inducing the formation of a DNA:RNA quadruplex. This supramolecular rearrangement into an intermolecular quadruplex results in an *in vitro* inhibition of reverse transcriptase activity (see Figure 6) (123). A similar approach was later used to target the mRNA of the human eukaryotic initiation factor (eIF-4E, whose overexpression is associated with cancer proliferation), resulting in decreased expression of the protein *in vitro* (124). The induction of the G4 on the mRNA transcript was indicated as the cause of the translation block (and therefore, of an increased level of eLF-4E mRNA), either by interference with the ribosome scanning or through the early interruption of protein synthesis leading to a truncated product and its subsequent proteolysis.

#### Targeting ‘G3’ sequences

Among the G-rich sequences which are not able to form G4 structures, the so-called G-triplex (G3)-sequences are of particular relevance (125). G3-forming sequences are equally abundant as G4-forming ones in the human genome and they can re-arrange into stable intermolecular quadruplexes when the missing guanine tract is added through an external probe. Indeed, G3 structures equipped with four third of the required G-runs are only able to form half of the Hoogsteen hydrogen bonds of a G4, and are, therefore, less prone to form under physiological conditions if the missing G-run is not present.

**Figure 7.**
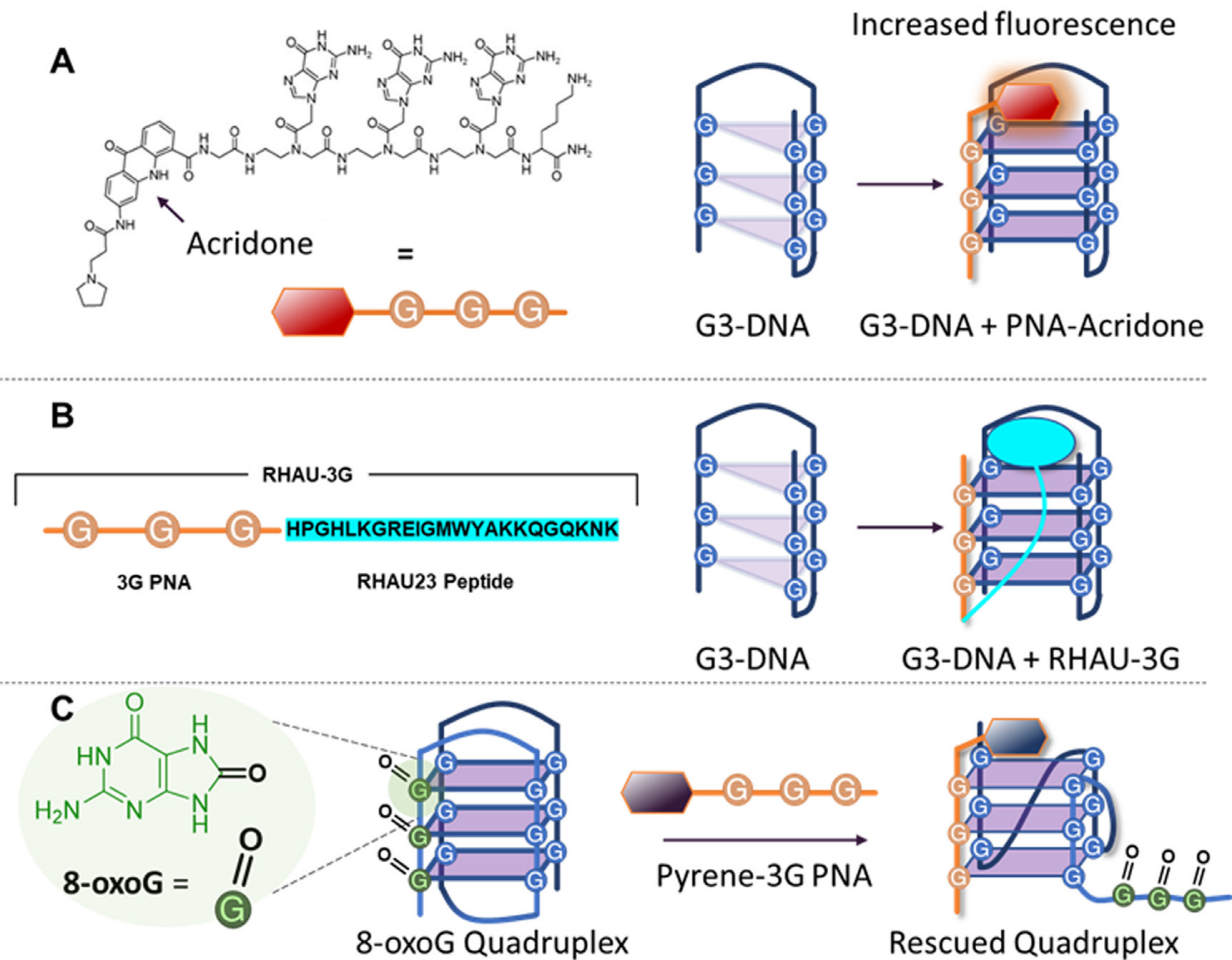
Examples illustrating the targeting of G3-sequences. (**A**) Schematic illustration of the methodology proposed by Ladame and colleagues, employing a 3G-PNA decorated with an acridone moiety (126). (**B**) Schematic illustration of the methodology proposed by Wen *et al.*, employing a 3G-PNA conjugated with the G4-binding peptide RHAU23 (131). (**C**) Restoration of a stable G4 structure from an oxidized 8-oxoG-containing structure (134).

To induce extra stabilization to the newly formed intermolecular quadruplex, extra modifications, such as G4-ligands, are often needed. Ladame and co-workers reported for the first time on the use of a G-rich PNA probe combined with a π-stacking G4 ligand (acridone), enabling the probe to form three tetrads with the three tandem repeats of the human telomeric sequence, while the ligand stacks on the outer tetrad, stabilizing the hybrid quadruplex in solution (Figure 7A). The choice of the acridone moiety additionally allows following the binding event via fluorescence: in the normal state, the PNA guanines quench the reporter fluorescence, while after binding to the target DNA the fluorescence is restored (126). Stabilization of such G4s was also achieved by conjugating G4-binding peptides rather than aromatic ligands to the targeting probes. From a synthetic point of view, the use of PNA is advantageous because of the straightforward coupling with additional ligands by solid phase peptide synthesis. Several proteins have shown the ability to efficiently bind and resolve G4 structures: BLM, FANCJ, DNA2, PIF1 and RHAU helicases, just to mention the principal ones (127–130). The binding domain of these proteins can be therefore used to design peptides with G4-binding properties. Accordingly, the 23-residue peptide fragment from the binding domain of RHAU (RHAU23) was exploited by Wen and colleagues to stack on top of the upper quartet of a PNA:DNA bimolecular quadruplex, thus stabilizing the construct. Noteworthy, the affinity for the targeted sequences proved up to 1000 times higher as compared to classic small ligands for their G4 targets, with *K*_d_s in the sub-nM range (131) (Figure 7B).

An extension of the concept of G3-targeting that deserves a special mention can be found in the work of Sugimoto and colleagues. Here, rather than targeting ‘proper’ G-lacking sequences, they describe the recovery of quadruplex formation starting from an oxidized G4 sequence in the promoter region of the *VEGF* gene, containing 8-oxo-guanines (8-oxoG) in place of regular guanines (Figure 7C). Because of its structure, 8-oxoG displays a preference for syn- rather than anti-conformation (132). Therefore, G4-sequences containing 8-oxoGs show a different, less stable, quadruplex topology and they are not recognized by nucleolin, a protein involved in quadruplex recognition and a putative responsible for the therapeutic effects of quadruplex stabilization (133). In Sugimoto's work, a 3G-PNA probe decorated with a pyrene moiety as G4-stacker is used. The guanines of the probe substitute the 8-oxoGs, thus inducing the formation of a more stable parallel intermolecular quadruplex containing only guanines in anti-like conformation. CD analyses and polymerase stop assays revealed the restoration of the original quadruplex topology as well as its biological functionality in terms of replication stall (134).

Besides the general lack of sequence-selectivity, the major drawback of inducing the formation of an intermolecular quadruplex using short G-rich tracks, is their self-assembly into intramolecular quadruplexes, especially when regular PNAs are employed. To solve this problem, the introduction of backbone modification appears essential. In this context, PNA oligomers bearing a γ-l-lysine side chain have been exploited for targeting G3 structures in human telomeric sequences while PNA-intramolecular quadruplex formation was not observed even at high strand concentration (135,136). Although this is not reported, in principle it would be possible to remediate the lack of sequence-selectivity by extending the probes to include one of the flanking regions of the quadruplex, thus orienting the missing G-track only where desired (*vide infra*). In principle, the use of this strategy combined with an accurate sequence-design can also allow avoiding the self-aggregation problem, as the formation of a stable PNA:DNA duplex would be favoured.

#### Targeting ‘4n-1’ sequences

**Figure 8.**
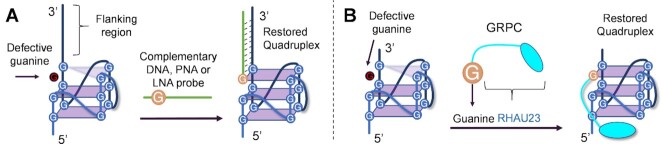
Examples of 4n-1 targeting strategies. (**A**) Sequence-specific targeting of 4n–1 sequence using a probe complementary to G4 flanking regions, as shown by Phan *et al.* (141). (**B**) Targeting of a guanine-deficient quadruplex, employing the G4-binding RHAU23 peptide linked to a guanine moiety (GRPC) (143).

A last approach towards the targeting of defective quadruplexes consists of targeting those sequences that lack only one guanine to form a stable quadruplex. They are formed by 4n-1 guanines (where n is the number of tetrads), therefore they assemble into less stable secondary structures as compared to the full G4s (137,138). Similar to G3-sequences, these ‘4n–1’ sequences are found to be as abundant as G4-forming ones, as ∼230 000 4n–1 sequences were estimated to be present in the human genome (139). Additionally, 4n–1 sequences could be generated as a result of single point mutations involving G4-forming sequences (140). Delivering the missing guanine (via an external oligonucleotide or mimic) allows restoring the correct biological function of the native quadruplex. Short 2–3mer PNA sequences already demonstrated the ability to fill the G-lacking site of 4n–1 sequences, delivering a remarkable contribution to the thermal stability of the G4 structure (Δ*T*_m_ ≈ + 20°C). However, the use of such short sequences did not result in sequence-selectivity. This was achieved by employing longer probes, designed to be complementary to the flanking region and facing the missing guanine, completing the last tetrad and forming the desired intermolecular quadruplex in solution (Figure 8A) (141). It is interesting to underline that, according to NMR studies, the three different probes used for the purpose (PNA, LNA and DNA) can lead to different results. Only the regular DNA probe led to the formation of a G4 structure in a single conformation. Alternatively, LNA led to the formation of a major conformation accompanied by the presence of a minor conformation, while in the PNA case a mixture of G4 and duplex formation was observed. These diverse outcomes were explained by the difference in the duplex-quadruplex junction or the formation of a probe:DNA intermolecular quadruplex. In addition, these differences might be sequence-dependent, and they could arise as a consequence of the different helical turns of the different duplexes. Although it was not evaluated, this comparative study very well underlines the differences that can arise when employing different oligonucleotide and mimics, from which the success of a targeting approach may depend. In a therapeutic context, if subsequent recognition of the formed G4 by a protein is needed (as shown above in the case of Sugimoto's work, depicted in Figure 7C), the employment of a DNA probe would be preferred. However, in case one aims at interfering with the expression of a target protein exploiting an anti-sense approach, the use of LNA probes or PNA analogues may be sufficient.

Single guanine residues linked to a G4-stabilizing agent can also be exploited to restore the quadruplex formation. This simple, but elegant approach was exploited by Tan and colleagues, who conjugated a guanine PNA monomer to the RHAU23 peptide (GRPC, guanine–RHAU23 peptide conjugate). This methodology benefits from a potent and synergistic action of the two members of this conjugate, which resulted in a high affinity at low nanomolar level. The designed GRPC showed a *K*_d_ which resulted 480-fold lower than the one reported for the commercially available pyridostatin, a small molecule considered as a ‘golden standard’ for G4-ligands (142). This work, however, has to be considered more as an extension of a small molecule-based targeting approach, rather than a proper sequence-selective targeting approach, as the specificity of this methodology remains limited by the capacity of recognition of the attached ligand/peptide (see Figure 8B) (143). However, with the introduction of a PNA-based guiding strand, following the principles described by Phan and colleagues in ref (141), the resulting approach would benefit from higher sequence-specificity, allowing to direct the selective formation of the desired G4-structure.

## INTERFERENCE WITH THE dsDNA-G4 EQUILIBRIUM: STRAND INVASION-MEDIATED G-QUADRUPLEX INDUCTION BY FORMATION OF HETERODUPLEXES

In the above-discussed modes of interaction, G-containing oligonucleotides, or mimics thereof, were shown to give rise to intermolecular quadruplex formation. As already discussed above, folding into a G4 normally occurs when the G-rich strand is not hybridized with its C-rich counterpart (64). In a cellular context, this can occur during DNA replication (when the two strands are separated in the replication forks), in the telomeres, and in certain tracts of RNA sequences. Therefore, a logical way to induce the formation of a desired quadruplex consists in the selective targeting of the complementary strand, which can then result in a free G-rich sequence able to undergo natural G4-folding. Also in this case, a common feature of the available G4-targeting methodologies exploiting strand invasion, is the use of oligonucleotide analogues, which are essential for achieving efficient strand displacement, as they can form more stable duplexes with the target DNA as compared to regular oligonucleotides.

In the simplest scenario, using standard DNA oligonucleotides, the strand invasion approach can be performed using a single G4-forming probe, which, in view of its compact G4-folding, displays better cell penetration and nuclease resistance than regular, non-G4-forming oligos. Once internalized, they behave like ‘regular’ anti-sense oligonucleotides, hybridizing to their C-rich counterpart and inducing the stabilization of the existing quadruplex in the sequence. Examples of DNA-G4 sequences employed in this context encompass the 27mer oligonucleotide derived from the c*-Myc* promoter gene (Pu27), which displayed sufficient uptake into leukemic cells to act as a cancer-selective antiproliferative agent *in cellulo* (144). Another example is given by the G4-forming sequence found in the promoter region of the vascular endothelial growth factor (*VEGF*) which, when hybridized to its C-rich counterpart, causes the downregulation of the growth factor, finding potential application in the treatment of cancer forms in which VEGF is upregulated, such as non-small cell lung carcinoma (145).

**Figure 9.**
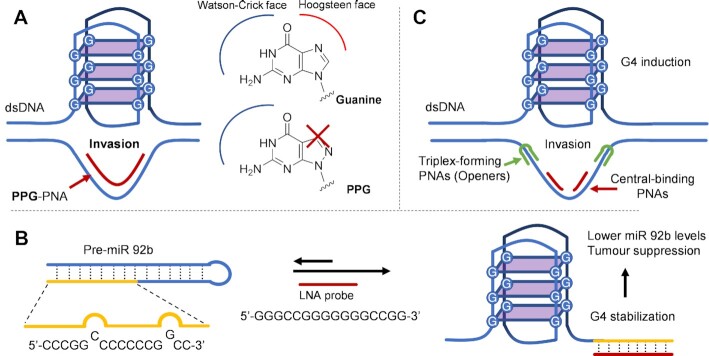
Strand invasion-based methodologies for targeting of G4-forming sequences. (**A**) Use of PPG–PNA to invade the BCL-2 proto-oncogene and avoid the formation of PNA intramolecular quadruplexes (146). (**B**) Targeting of pre-miR-92b by blocking the C-rich portion of the pre-miR hairpin (149). (**C**) Inducing G4 formation in plasmid DNA, using two different probes for a) opening the target dsDNA (triplex-forming PNAs, in green) and b) forming a stable duplex with the C-rich strand (Central-binding PNAs, in red) (150).

When using PNA probes for such purpose, it is crucial to avoid their aggregation into homo- or intermolecular quadruplexes, so careful sequence-design is necessary. Additionally, chemical modification of the employed probes can be useful. As an example, PNAs modified with pyrazol(3,4-d)pyrimidine guanine (PPG, a guanine derivative where the N7 nitrogen is shifted to position 8, showed the ability to invade the *BCL-2* sequence and induce G4-folding of the freed G-rich strand (Figure 9A). The absence of the nitrogen in seven position prevents the formation of Hoogsteen interactions, without affecting the ability to form Watson–Crick interactions. As a result, no self-aggregation of these PNAs can occur, while the ability to recognize the complementary cytosine is maintained (146).

Examples of strand-invasion do not only concern DNA structures. Indeed, as mentioned in the introduction, the formation of G4-structures has also been reported for non-coding RNA sequences, such as miR and miR precursors. Basu *et al.* reported on the induction of G4-formation in the human pre-miR-92b hairpin, whose mature miR is responsible for the suppression of the oncosuppressor protein PTEN and connected to resistance to chemotherapeutics (147). G4-formation in the pre-miR was found to play an important role in the maturation process by disrupting the canonical stem-loop structure and inhibiting DICER recognition (148). The delicate equilibrium between hairpin and quadruplex depends on cellular K^+^ levels, but it was found that LNA probes are able to invade the hairpin-structure of pre-miR-92b, inducing G4-formation and causing a reduction in miR-92b levels in the cell (Figure 9C). Although it does not appear clear whether the effects are due to the formation of a G4 structure or a mere anti-sense approach, the G4 formation appeared essential to guarantee optimal strand invasion by the LNA probe. Indeed, in absence of K^+^ the strand invasion was not successful (149).

Strategies involving the use of multiple probes have also been reported. The success of such a multiple-probe approach was demonstrated by evoking intramolecular quadruplex formation in the *BLC-2* plasmid DNA (Figure 9C). A triplex-forming PNA strand was used to target a polypyrimidine region adjacent to each side of the quadruplex-forming sequence, inducing the opening of the double stranded DNA and rendering the C-rich region accessible for the second, central-binding PNA probe. This simultaneous binding of the two PNA probes resulted in strand displacement, enabling the subsequent folding of the G-rich sequence (150). Additionally, the influence of the PNA charge was investigated. Due to the electrostatic repulsion of the target, a double negatively charged PNA displayed no invasion of the plasmid DNA, compared to the double positive and zwitterionic PNAs, while the latter displayed the highest sequence-specificity (151). This last point very well highlights the importance of the PNA charge, required for solubilizing the probe, and from which the success of this targeting approach may depend.

The examples reported above demonstrate that the C-rich strand can be successfully exploited for designing sequence-selective approaches, as all the complementary elements of the whole quadruplex (G-runs, loops, and flanking regions), can be targeted by the same probe sequence. On the other hand, the major limitation resides in the G-rich character of the probes employed for the purpose, which can lead to self-aggregation, especially when a PNA analogue is employed. It was demonstrated that this problem can be overcome by using modified nucleobases (146), but as described above for the intermolecular quadruplex formation, also the use of backbone-modified probes with stereocenters, such as γ-PNAs, although not reported in this precise context, could show promise. Alternatively, an idea to by-pass the potential self-aggregation can be to extend the length of the probe, to target the flanking regions, provided they do not contain a C-stretch. In this way, the relative G-content of the PNA is lowered and, at the same time, the stability of the resulting duplex can be increased.

## G-QUADRUPLEX ARCHITECTURE DISRUPTION

The gene-regulating effect of G4s in promoter regions of the DNA and the ability of RNA-G4s to enhance mRNA translation indicate the rationale behind the idea to interfere with the G4-formation. However, the implications of disrupting these structures, abundant in the 5′-UTR regions, are not entirely understood (152,153). Even though more studies are needed to understand the therapeutic significance of disrupting (or invading) DNA-G4s and RNAs, sequence-specific disruption offers an enormous opportunity to understand the biological role of a G4. As we already discussed in previous sections, G4-formation tends to occur when the two complementary strands are separated. Therefore, to disrupt the G4-architecture one can design a complementary C-rich probe, able to form a tight double-stranded structure (quadruplex invasion).

**Figure 10.**
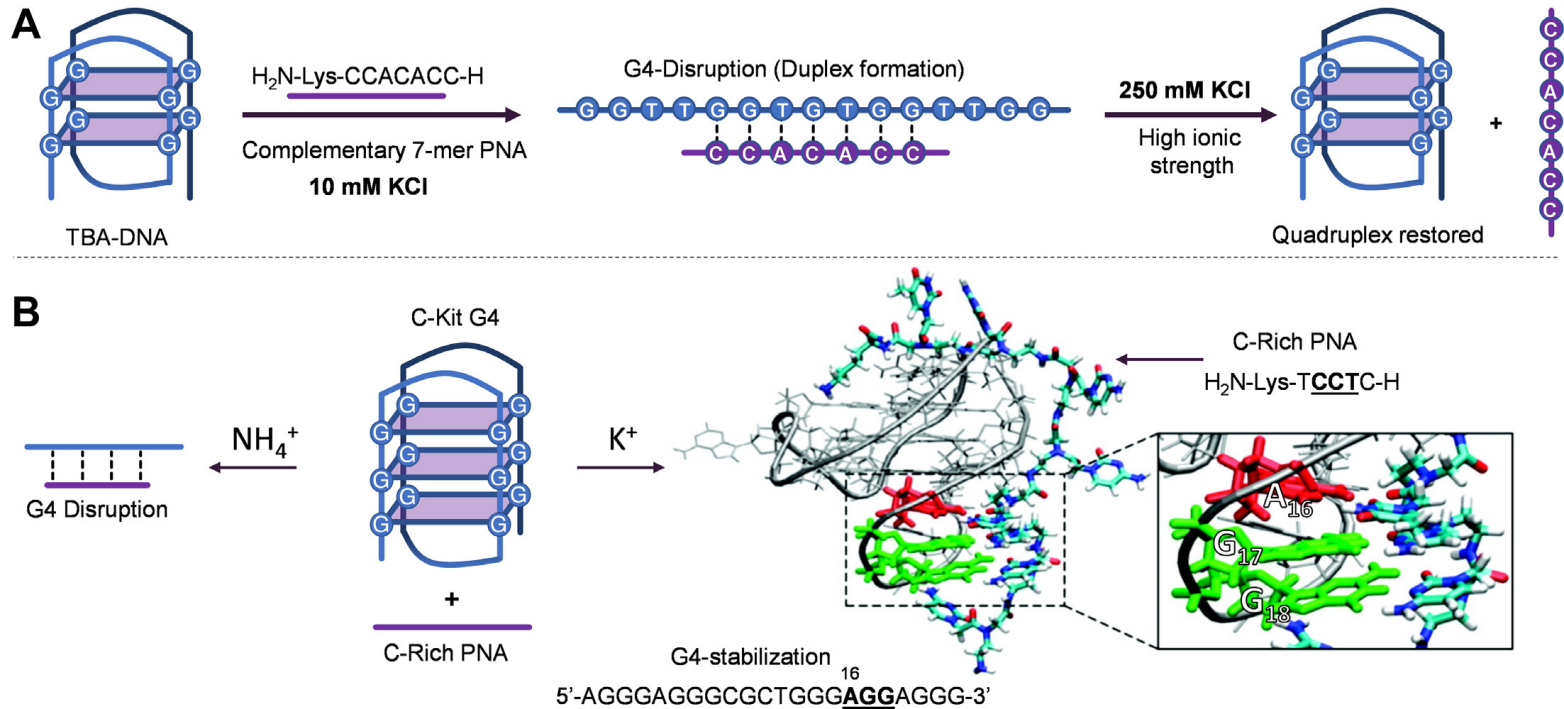
Example of approaches for G4-disruption, mainly involving the targeting of the central bases of the quadruplex. (**A**) Targeting of TBA G4 by using a 7-mer PNA probe and restoration of G4 at high K^+^ concentration, as shown by the Armitage group (155). (**B**) C-Kit targeting by a complementary C-rich PNA, by Amato *et al.*, which shows a different behaviour depending on the buffer used (157).

Pioneering studies on G4 invasion revealed that G4-folding can be disrupted even using a single probe, designed to target the key structural features involved in G4-stability, such as the central G-runs and loops (154). Armitage and co-workers reported on the unfolding of the thrombin binding G4 aptamer using a 7-mer PNA, complementary to the central tetrads and loop. It was found that the duplexes formed upon PNA hybridization were more stable when overhangs from unpaired DNA are present. This was attributed to an ‘overhang stabilization’ effect in which the extra bases can stack onto the duplex and contribute to complex stability. As this is the result of the different structure of the resulting, less tight (13 versus classical 10 basepair per turn), helical PNA:DNA duplex (155), this extra stabilization would not be possible using natural oligonucleotides. Additionally, as a consequence of the small influence of ionic strength on PNA:DNA duplex stability, it was also shown that restoration of the G4 structure could be obtained by increasing the K^+^ concentration (Figure 10A). The same group compared the effect of the hybridisation of either homologous (oligomers having the same G-rich sequence as the target, prone to induce the formation of a intermolecular quadruplex) or complementary (C-rich sequences which target the quadruplex forming a duplex) 7mer PNAs. While the homologous probes can lead to four different DNA_2_:PNA trimolecular quadruplexes, with a topology depending on the orientation in which the PNA binds the target (112), the complementary probes induce heteroduplex formation, with subsequent disruption of the G4-folding (111).

Conformationally different G4s can respond variably when targeted with either complementary or homologous probes. As discussed before (please refer to section 3.1), the parallel G4 formed by the *c-Myc* sequence and containing only anti-oriented guanines, is more prone to form intermolecular quadruplexes with a homologous probe at high K^+^ concentration, while the quadruplex disruption with the complementary probe is discouraged. However, in presence of NH_4_^+^ buffer, the intermolecular quadruplex formation is impaired. In contrast, in K-rich buffer, the hybrid G4 formed by h-Telo does not rearrange into an intermolecular quadruplex when using a homologous probe, but it readily forms a PNA:DNA duplex when using a complementary probe (156). Although more studies are needed to draw conclusions, this additionally confirms that parallel quadruplexes are more difficult to invade and a targeting approach involving the formation of intermolecular quadruplexes offers more chances of success.

As it emerged from these studies, the type of buffer and the ionic concentration may have a big impact on the targeting strategy used (149,155,156), as confirmed in later comparative studies. This factor needs to be taken into account when designing bench experiments. In fact, when using an NH_4_^+^-rich buffer, the PNA probes containing cytosine-tracts complementary to c-Kit 87′s central tetrad and loops were able to disrupt the quadruplex architecture, while this was not possible in K^+^-containing buffers (157) (Figure 10B).

It was already mentioned in section 3.1 that modification in γ position of the PNA backbone can be exploited to lower the affinity for a complementary strand and maximize intermolecular quadruplex formation. However, in the context of G4-disruption, similar modifications can be expected to increase the affinity for the complementary probes to induce duplex formation. This strongly depends on the handedness of the pre-organized PNA helix, whose orientation is determined by the chirality at the γ position, and by the steric hindrance given by the presence of the extra modification. Right-handed helical probes (obtained with γ-L-PNA-modified backbones, e.g. γ-L-Ala or γ-L-Ser) showed higher affinities for the complementary targets as compared to regular PNAs (158). As a result, γ-PNAs revealed to be more potent and selective in invading RNA-G4s as compared to regular PNAs and 2′-OMe-RNAs. Luciferase reporter assays, used to evaluate translational inhibition caused by the hybridization of a PNA with its target, revealed that the highest effects were obtained when the quadruplex in 5′-UTR was targeted together with their adjacent flanking regions (159).

Thanks to the higher thermal stability of their heteroduplexes, PNAs are largely used for disrupting G4 architecture and, therefore, are preferred over other derivatives, where the physiological concentration of K^+^ can make the G4 sufficiently stable to make the invasion approach problematic. There are however few examples where LNA probes were successfully applied for quadruplex invasion. Examples are given by the selective targeting of *c-Myc* G4 using a probe complementary Pu27 sequence (93), or by the disruption of the RNA-G4 in the 5′-UTR of H2AFY encoding sequence with LNAs and 2′*O*-Me RNAs, modulating its expression *in cellulo* (91). This last application would be particularly useful in a diagnostic context, as the H2AFY protein is a relevant cancer biomarker correlated with tumour dissemination and metastasis.

Depending on the length of the central loop targeted, the approach described can reach considerable selectivity levels. Applications foreseeing this strategy have successfully been applied in a cellular context to target mRNA of interest, demonstrating the high therapeutic potentiality of anti-sense approaches relying on RNA-G4 targeting. Also in this context, the use of PNAs is largely preferred over other analogues. However, the targeting of short-looped quadruplexes is, in our opinion, the major limiting factor for the application of this methodology, as it drastically diminishes the sequence-variability, limiting the selectivity of the approach. To solve this issue, one could simply extend the probes in 3′ or 5′ direction. Although there are no examples reported, we speculate that the use of probes complementary to the one of the flanking regions of the target, equipped with ligands able to disrupt the G4-folding (81), can be a helpful solution in this context, as the probe employed would not be limited by the loop composition.

## G4-STABILIZATION AND OTHER APPROACHES NOT INVOLVING INTERMOLECULAR QUADRUPLEX FORMATION: TARGETING QUADRUPLEX LOOPS AND FLANKING REGIONS

As illustrated with the previous examples, several methodologies can allow a satisfactory G4 sequence-specificity. A C-rich probe can be used when disruption of the targeted structures is desired, while, on the other hand, a G-rich strand can guarantee double-strand invasion to enhance the ‘natural’ G4-formation. An alternative approach for increasing the stability of an existing G4 can consist in targeting the other important structural elements of the quadruplex, namely the loops and the flanking regions, which contribute to making each G4 unique in the whole genome. Given the uniqueness of the loop and flanking sequences, approaches based on a direct interaction with these structural elements are the most promising to develop sequence-selective methodologies.

**Figure 11.**
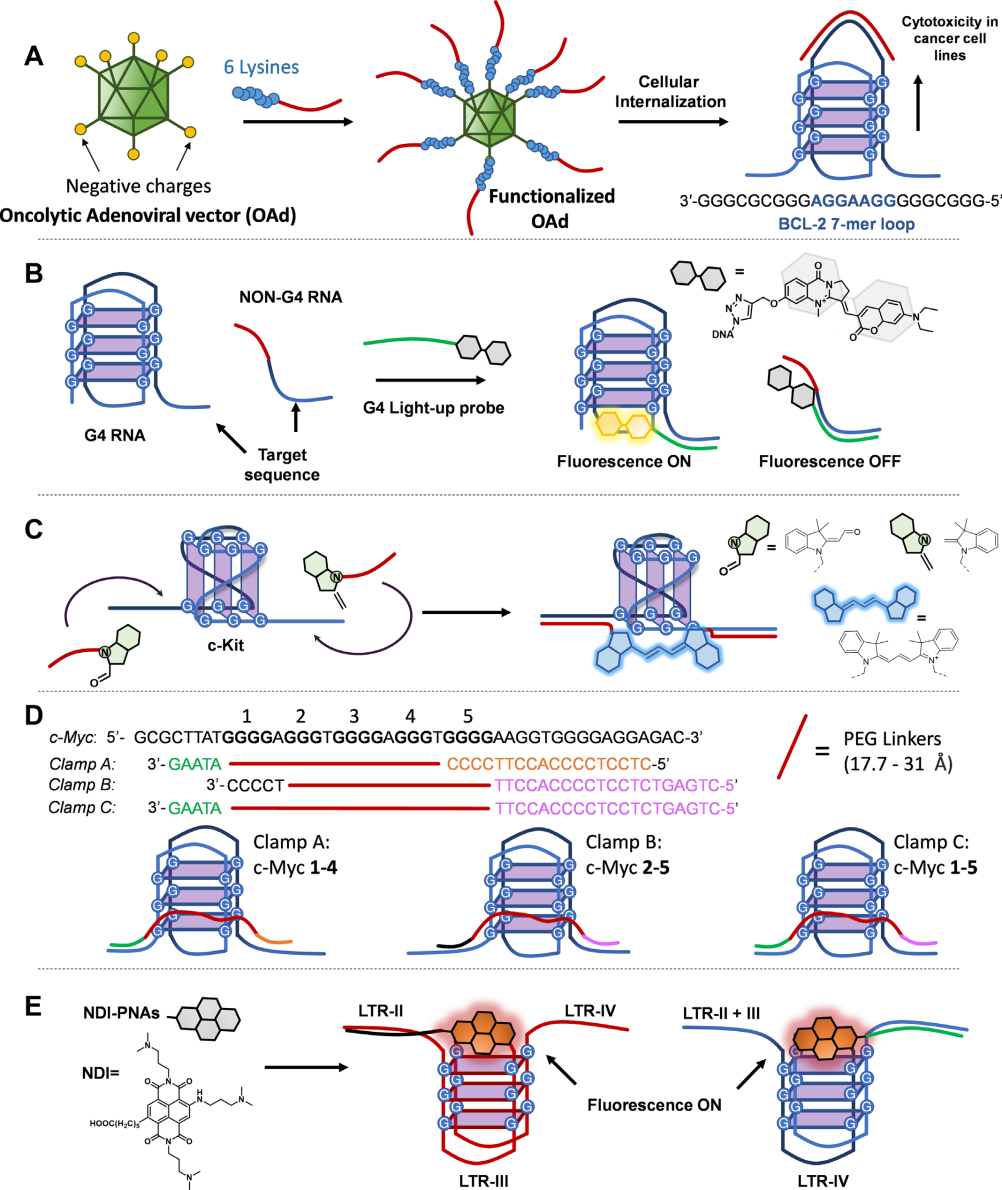
Examples of approaches for G4-targeting exploiting either loops or flanking regions. (**A**) Targeting of the BCL-2 long loop in cells, exploiting vehiculation through an adenoviral vector, as shown by Amato *et al.* (160). (**B**) Approach shown by Chen *et al.*, for G4 detection using a turn-on fluorescent dye approach (162). (**C**) G4-templated synthesis of cyanine fluorophores, described by Ladame and co-workers (164). (**D**) c-Myc targeting using a clamp-based approach as proposed by Hao *et al.* (166). (**E**) Targeting of mutually-exclusive G4 sequences in the HIV-LTR genome, as proposed by Richter and colleagues (168).

As discussed in the previous section, Amato *et al.* illustrated control over the expression of the c-Kit gene by targeting the loops and central G-tracks of *c-Kit* 87 in a K^+^ solution with complementary PNA probes (157), obtaining an opposite effect in presence of NH_4_^+^-rich buffer. In the same work, they showed that when using a PNA complementary to the 5mer loop of c-Kit, the stabilization of the G4 structure occurred even in presence of NH_4_^+^, as the PNA acted as a quadruplex-stabilizing agent (Please refer to the previous Figure 10B). Recently, the same group reported on the use of a short 7-mer PNA for targeting the central 7-bases long loop of the promoter of the mitochondrial protein BCL-2 involved in apoptotic processes. The positively charged PNAs containing a 6-lysine stretch were successfully delivered inside the targeted tumour cells using an oncolytic adenoviral vector (OAd) able to recognize CAR receptors, whose negatively charged capsid could be decorated through electrostatic interactions. The OAd, was able to selectively internalize the cargo through the recognition of CAR (overexpressed in cancer cell lines), achieving good cellular internalization and cytotoxic effects. (Figure 11A) (160).

In general, due to the limited length of the loops in G4 structures (1–7mer); approaches of this kind require the use of oligonucleotide analogues rather than natural nucleotides, owing to the higher stability of the complexes formed. The length of the loop has important consequences in terms of selectivity, as the number of G4s that can be targeted using longer probes is more limited (the aforementioned BCL-2, or the target sequences described in (114)).

Other important structural elements that can be exploited to stabilize and target a G4 are represented by the flanking regions at the 3′- or 5′-end of the sequence. The use of probes complementary to the flanks offers a specific advantage, as their length can be indefinitely extended for increasing the sequence-specificity and the duplex stability, allowing the use of regular oligonucleotides rather than the more expensive analogues. The approach is currently used for imaging the target G4 upon internalization of fluorescent probes and, most importantly, for designing novel theragnostic platforms based on quadruplex formation, allowing the simultaneous visualization as well as targeting of the desired sequence. This concept was successfully employed for visualizing *NRAS* G4 through the hybridization of an oligonucleotide bearing a turn-up dye, to the 3′-flanking region of the target, both *in vitro* and *in cellulo*. When the probe binds the target, the ligand exhibits a fluorescence turn-on, due to the end-stacking interactions with the external G-tetrad (161,162) (Figure 11B). A similar tactic was described for the theragnostic targeting of human telomeric DNA sequences, and the G4-stabilization effect demonstrated with both PCR stop assays and CD analyses (163). The simultaneous targeting of both flanking regions can also be exploited to bring two targeting probes in close proximity, for the realization of G4-templated ligation reactions, used for target detection. Ladame and co-workers used c-Kit 21 as a template for a fluorogenic aldol condensation between two complementary PNA probes bearing an indoline and an aldehyde function. The resulting fluorescent cyanine allowed target sensing down to 500 nM (Figure 11C). Although the group did not report on additional stabilization of the G4 target induced upon ligation, we can classify this as a theragnostic application, as extra stabilization of the target can be expected after the formation of the ligation product (164,165).

A last application allowed by targeting the G4 flanking regions consists in locking the sequence of interest into a particular isoform. As mentioned in the introduction, particularly long sequences containing several consecutive G-runs have the possibility to form multiple isoform G4-structures featuring the participation of different G-runs, which may exhibit different topologies. In a work by Hao *et al.*, a DNA-clamp approach was exploited to target the *c-Myc* NHE-III region, inducing the formation of the desired G4 isoform (Figure 11D) (166). Oligonucleotide sequences were designed to display two fragments, complementary to both flanking regions of the targeted sequence, connected via PEG-based linkers of various lengths. They showed the possibility to lock the conformation of the target into the desired isoform, depending on the length of the spacer and the clamping sequence used. They further demonstrated that amongst all the possible structures that can be formed within *c-Myc* promoter region, only one of them, the one involving G-runs 1–4 and predominant under physiological conditions, is responsible for gene downregulation, demonstrated via *in cellulo* transfection HEK-293 cell lines (166,167).

Isoform induction was also recently achieved using a single PNA probe decorated with a small G4-ligand targeting the HIV-LTR region. In the work proposed by Richter and co-authors, the PNA has the double role of bearing the ligand, a non-selective naphtalenediimide (NDI) end-stacking binder, in proximity to the desired G4-isoform, blocking at the same time the other overlapping structures through Watson-Crick base-pairing. On the other hand, the choice of NDI as ligand, allowed a fluorescence turn-on when interacting with the external tetrad, yielding an interesting theragnostic application (168) (Figure 11E). Interestingly, the designed NDI-PNAs proved to be co-localized with G4–DNA in a cellular model.

As compared to the approach based on loop-targeting, methodologies exploiting the recognition of flanking region, in principle, have a greater potential, as all G4-forming sequences can be targeted using these approaches, without boundaries set by the type of target. In addition, there are no limits concerning the length of the sequence that can be targeted to reach satisfactory duplex stability and, therefore, even regular oligonucleotides can be used in the context.

Finally, the relatively unexplored field of the possibility to induce the formation of the desired quadruplex isoform within the same sequence holds great therapeutic potential. So far, all the reported strategies for switching among possible G4-isoforms exploited the hybridization of the external G-runs and the adjacent flanking region. However, similar approaches could be, in principle, used to block central G-runs, inducing the formation of unnatural quadruplex isoforms This of course can only be possible if the number of bases in the loop flanking the target G-run is sufficiently long to ensure stability and selectivity. In this context, one could in principle induce the formation of less stable quadruplexes bearing a longer loop, normally unexpressed in physiological conditions. Although this approach, to our knowledge, was not tested, we believe that it can be interesting for studying the biological role and targeting significance of these alternative G4-isoforms.

## POTENTIAL EXPLOITATION OF G4-FORMING OLIGONUCLEOTIDES IN THERAPEUTIC APPLICATIONS

The presence of preserved G4s in the promoter region of proto-oncogenes and the feasibility to regulate gene expression following the discussed modes of action emphasizes the importance of selectively targeting a specific sequence. Despite not directly falling under the classification of G4-targeting approaches, there are other potential therapeutic applications relying on the use of G4-forming oligonucleotides *per se*, which need a special mention, as they have *in vivo* applications and have reached clinical trials.

An example is given by those G4-forming nucleotides that, once folded, can recognize a specific structure, such as a protein domain. The mechanism of action obviously depends on the targeted protein, but in most cases involves binding to the target structure and the subsequent interference with its normal function. Approaches of this kind, exploiting a decoy strategy, are illustrated with the use of synthetic analogues mimicking *HRAS-1* and *KRAS* quadruplexes (169,170). This strategy relies on the binding of this Ras analogue to the nuclear transcription factor MAZ, thus reducing its association with Ras promoter, leading to reduced transcription of the gene in mice (171). To increase the efficiency of this approach, DNA–LNA chimeras decorated with a π-π stacking anthraquinone moiety were exploited (172).

A common target of G-rich oligonucleotide sequences is nucleolin, a eukaryotic multifunctional phosphoprotein involved in protein transportation, cellular replication, ribosomal packaging, and telomerase activity and which features high expression levels in cancer. An anti-nucleolin aptamer, the G4-forming oligonucleotide AS1411, is in clinical trials for treatment of cancer (173). AS1411 is probably the most famous example available in literature; however, several G4-forming oligonucleotides have been identified, through systematic evolution of ligands by exponential enrichment (SELEX), as aptamers for a selected target. In this context, the application of RNA-based aptamers, capable of recognizing RNA-G4s over other secondary structures is worth mentioning. Through tight complexation of the G4–target, the interactions with RNA–G4 binding proteins such as nucleolin (174) or RHAU (33) are blocked, thus resulting in a potential therapeutic effect. As for the case of small-molecules binders, this topic has already been elaborately described, therefore we will not consider it in detail here and we refer the reader to the most recent reviews that have been published on this subject (175–177). Another potential application of G4-forming oligonucleotides is based on the exploitation of their peculiar pharmacokinetic properties in terms of better cellular internalization and nuclease-resistance. In this context, G4-forming RNAs are studied as potential delivery systems for small molecules, displaying good internalization in cancer cell lines (178).

## CONCLUSIONS AND FUTURE PERSPECTIVES

Since their first discovery, the scientific attention for G4s has only continued to grow. This has led to the discovery of hundreds of small-molecule-based ligands over the past decades. However, even if displaying high selectivity for G4 over dsDNA, most of them do not exhibit sequence-selectivity for a specific G4 sequence and, as a direct consequence of the ubiquitous presence of G4-forming sequences in the human genome, this may lead to undesired interactions. The selectivity is, in our opinion, an absolute requirement for G4-targeting applications to allow moving beyond the lab benches in which, in most cases, they stay confined to date.

In this review, we have pointed out the importance of a recognition element to achieve the so-desired sequence-selectivity, highlighting several different strategies in which synthetic oligonucleotides and mimics thereof target G4s in a sequence-selective fashion, increasing the potential applicability in therapeutic contexts. Additionally, by developing such selective methods, valuable tools to study and better understand the specific role of the single G4 of interest are provided, thus significantly increasing our target-identification ability.

In general, the most promising approaches, which allow sequence-specific targeting, involve loops and, even more, flanking regions as targets, as their more varied composition is key for increasing the sequence-variability. Due to the long extension of the flanks, the probes used for reaching selectivity can be prolonged, with the ‘sole’ limitation given by the cost of long oligonucleotides.

On the other hand, we identified the limits of other methods, such as those relying on the induction of intermolecular quadruplexes *per se*, that still need further optimization for reaching a similar selectivity level and are, in our opinion, less promising. Nevertheless, we think that the latter would sensibly benefit from the integration of elements of other strategies, such as the use of a flanking region to direct the probe to the desired G4, the use of complementary strand-invasion methodologies, and even the attachment of G4-ligands on the probes employed. Approaches of this kind will be time-consuming, requiring hefty design and synthetic efforts. In the future, it will be interesting to integrate the knowledge that we have on the structural properties of G4s, on the binding mode of the available ligands and on the properties of the synthetic oligonucleotides analogues, to develop modelling-assisted approaches for reducing the efforts and direct the researchers in the right direction.

To conclude, the approaches described in this review represent promising alternatives to small molecules in the context of G4-targeting. However, even once the required selectivity would come within reach, challenges will remain:

- What are the consequences of targeting a specific G4 involved in the physiological regulation of specific pathways? Thanks to the ‘ideal’ sequence-selectivity, one would be able to affect the formation of the desired RNA or DNA–G4, thus increasing the knowledge relative to the gene/transcript of interest. Selective approaches could be used in this context as a tool to investigate the role of these specific structures in the fine regulation of cellular pathways.
- Which of the described strategies offer genuine potential in a therapeutic context? Many of the described systems can be applied in a diagnostic context (162–165,168) or to study the properties of a specific G4. However, when it comes to in vivo applications, only some of them have been tested in cellular models (91,93,149,160,162,166,168) and even fewer of them have been successfully applied in animal models (170,173). When considering these systems in detail, it can be noticed that the ones tested in vivo mostly feature the use of standard oligonucleotides or LNA probes, while most of the applications relying on the use of PNAs are still far from close to a genuine application in this context. This can likely be traced back to the intensive research efforts in the development of delivering methodologies for oligonucleotides featuring negatively charged backbones as compared to other mimics, resulting in well-established vehiculation techniques available nowadays for those derivatives.
- How to effectively deliver an oligonucleotide analogue construct not only in vitro but also *in vivo*? An inherent problem connected to the use of oligonucleotides as therapeutics resides indeed in their difficult in vivo vehiculation into the tissue of interest. The need for extrahepatic localization imposes the use of efficient delivery methods to enhance their permeation in the desired tissues and is still (one of) the major limitation(s) for oligonucleotide-based therapeutics. In the work described in the current review, the main transfection methodologies used for cellular internalization rely on lipofectamine (91,149,162) and plasmid transfection (93,166), although examples of delivery via adenoviral vectors (160), or through constructs with inherent internalization abilities (168) are also reported. On the other hand, PNA analogues, while devoid of a negatively charged backbone, are strongly affected by endosomal and lysosomal entrapment, needing special care to address a higher effective cellular concentration of these probes. We refer the reader to other available reviews for a more complete overview of methodologies for efficiently delivering oligonucleotides and analogues in cells (179,180). Although additional investigations on how to obtain better cellular penetration are required, we strongly believe that the promising potential of these strategies in cancer-cells and viral infection models makes the research efforts worthwhile. For a genuine clinical application, delivering the construct in the tissue of interest and a good internalization will be key factors still requiring considerable research efforts.’
- Will it be possible to achieve effective sequence selectivity using covalent methodologies, introducing examples of sequence-specific alkylation of quadruplexes? Currently, all the approaches discussed in this review are based on non-covalent interactions between the probes and the targets. Recent examples exist of methodologies for covalent interaction with G4–DNA but, because they rely on the use of small molecules, they are not exempt from possible off-target interactions. The use of bi-molecular approaches, relying on the contemporary presence of two G4-binders, can help in this context by increasing the selectivity over double-stranded DNA, although genuine sequence selectivity remains an issue to solve (181). Given the high level of selectivity that can be reached using sequence-recognition approaches, we foresee future studies in this direction.

With the crescent understanding of quadruplex biology and their regulatory aspects in the whole transcriptome, we expect that the sequence-specific targeting of RNA-G4 will be the next step to achieve in a biological model, as the cytosol will be easier to reach as compared to the cell nucleus. This will significantly increase our understanding of the biological pathways regulated by RNA-G4s, whose *in vivo* role is, today, still not entirely clear (182).
